# Supplementary material for: Enhancement of Human Immunodeficiency Virus-Specific CD8+ T Cell Responses with TIGIT Blockade Involves Trogocytosis
Source: Pathogens. 2024 Dec 23;13(12):1137. doi: 10.3390/pathogens13121137 (PMC11679564; doi:10.3390/pathogens13121137)
Supplement: Supplementary file 1 [file pathogens-13-01137-s001.zip › pathogens-3341821-supplementary.pdf]

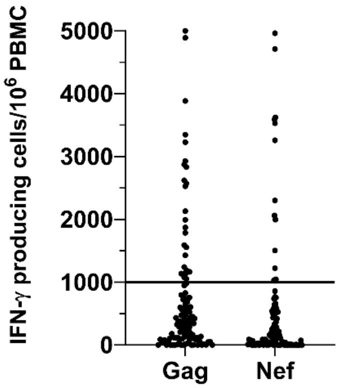

Figure S1. Measurement of HIV Gag- and Nef-specific T cell responses in PLWH. Antigen-specific T cell responses were measured by IFN-γ ELISpot with PBMC from PLWH exposed to full sets of overlapping peptides spanning HIV Gag or Nef at 1 µg/mL each peptide. The horizontal line in the graph indicates our chosen threshold for T cell response magnitudes suitable for further study by flow cytometry.

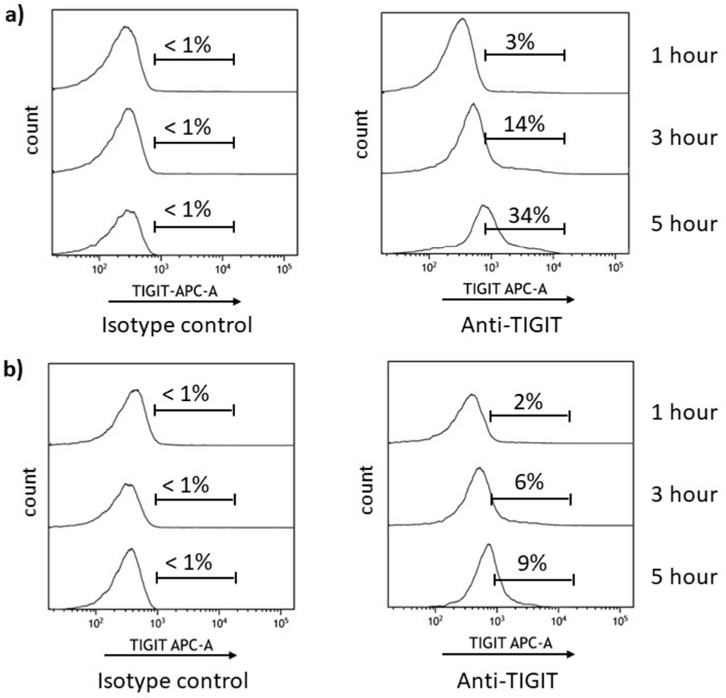

Figure S2. Measurement of gain in anti-TIGIT mAb fluorescence on monocytes with *in vitro* TIGIT blockade over 5 hr incubation. Freshly-isolated PBMC from 2 PLWH (a, b) incubated with fluorescence-conjugated isotype control or anti-TIGIT mAb were harvested at 1, 3 and 5 hours and anti-TIGIT mAb fluorescence on monocytes meaured by flow cytometry. Gating on monocytes was as in figure 4. Results with isotype control (left hand plots) and with anti-TIGIT mAb (right hand plots) after one, three and five hours are shown with per cent monocytes positive for anti-TIGIT fluorescence indicated above the markers.
